# Gene-sex interactions in schizophrenia: focus on dopamine neurotransmission

**DOI:** 10.3389/fnbeh.2014.00071

**Published:** 2014-03-06

**Authors:** Sean C. Godar, Marco Bortolato

**Affiliations:** ^1^Department of Pharmacology and Toxicology, School of Pharmacy, University of KansasLawrence, KS, USA; ^2^Consortium for Translational Research on Aggression and Drug Abuse, University of KansasLawrence, KS, USA

**Keywords:** schizophrenia, dopamine, catecholamine-O-methyltransferase (COMT), monoamine oxidase (MAO), gene-sex interactions, sex hormones

## Abstract

Schizophrenia is a severe mental disorder, with a highly complex and heterogenous clinical presentation. Our current perspectives posit that the pathogenic mechanisms of this illness lie in complex arrays of gene × environment interactions. Furthermore, several findings indicate that males have a higher susceptibility for schizophrenia, with earlier age of onset and overall poorer clinical prognosis. Based on these premises, several authors have recently begun exploring the possibility that the greater schizophrenia vulnerability in males may reflect specific gene × sex (G×S) interactions. Our knowledge on such G×S interactions in schizophrenia is still rudimentary; nevertheless, the bulk of preclinical evidence suggests that the molecular mechanisms for such interactions are likely contributed by the neurobiological effects of sex steroids on dopamine (DA) neurotransmission. Accordingly, several recent studies suggest a gender-specific association of certain DAergic genes with schizophrenia. These G×S interactions have been particularly documented for catechol-O-methyltransferase (COMT) and monoamine oxidase (MAO), the main enzymes catalyzing DA metabolism. In the present review, we will outline the current evidence on the interactions of DA-related genes and sex-related factors, and discuss the potential molecular substrates that may mediate their cooperative actions in schizophrenia pathogenesis.

## Introduction

Schizophrenia is a chronic and severe neurodevelopmental disorder, characterized by a highly complex and heterogeneous set of perceptual, cognitive and emotional deficits (Breier, 1999; Rowley et al., 2001). According to the current diagnostic criteria, the pathognomonic manifestations in schizophrenia are clustered into three groups of symptoms: (1) *positive symptoms*, which encompass hallucinations and delusions; (2) *negative symptoms*, including flat affect, alogia, anhedonia and social deficits; and (3) *cognitive symptoms*, which reflect impairments of attention, memory, perception and thought. Converging evidence has revealed that the primary deficits in schizophrenia are likely mediated by dopamine (DA), in cooperation with other key neurotransmitters, such as glutamate, γ-aminobutyric acid (GABA) and serotonin. Nevertheless, the quest to understand the pathogenic mechanisms of schizophrenia has not yet led to a conclusive theory, and its pathophysiology remains frustratingly elusive.

A wealth of genetic data has identified a number of vulnerability factors that are not inherently pathological, but predispose an individual to develop schizophrenia in the presence of critical environment determinants. These findings have prompted a shift in the conceptual framework of schizophrenia, and underscored the importance of gene-environment (G×E) interactions in this disease (Van Os and Murray, 2008; Van Os et al., 2008, 2010; Van Os and Rutten, 2009).

Multiple lines of evidence have also highlighted that sex-related factors play a potentially important role in shaping the clinical trajectory of schizophrenia. Indeed, males have a higher risk for schizophrenia than females, with earlier age of onset and greater severity of negative and cognitive symptoms (Markham, 2012). Based on these premises, it is possible to theorize the existence of specific gene × sex (G×S) interactions that may also contribute to schizophrenia pathogenesis.

Numerous preclinical studies support that the DAergic system is one of the key mediators of sex differences in schizophrenia (Bay-Richter et al., 2009; Arime et al., 2012; for a detailed presentation of this issue, see Sanchez et al., 2010); accordingly, genetic investigations point to a clear involvement of the key metabolic enzymes of DA, catechol-O-methyltransferase (COMT) and monoamine oxidase (MAO), in the underpinnings of G×S interactions in schizophrenia. In the present review, we will discuss how the emerging evidence on the genes encoding these enzymes and their interactions with sex-related factors may provide fundamental clues to unravel the essence of the biological bases of schizophrenia.

## The role of DA in the pathophysiology of schizophrenia

The role of dopamine in the pathogenesis of schizophrenia was originally postulated following the discovery that D_2_ dopamine receptor antagonism was a fundamental pharmacological requisite of antipsychotic drugs, and that the therapeutic efficacy of these agents was correlated with their inhibitory potency (Seeman and Lee, 1975; Creese et al., 1976). While several studies support the concept that stimulation of D_2_ receptors in subcortical areas (and particularly striatum and nucleus accumbens) results in psychotic manifestations, other lines of evidence strongly suggest that negative and cognitive symptoms (which are generally not affected by D_2_ receptor antagonists) may be underpinned by the insufficient activation of D_1_-like receptors in the prefrontal cortex (PFC) (Goldman-Rakic and Selemon, 1997). These findings have led to the view that schizophrenia may be underpinned by mesolimbic hyperactivity and mesocortical hypoactivity (Weinberger, 1987; Davis et al., 1991). Although studies in the last two decades have documented the fundamental roles of other neurotransmitters in schizophrenia, particularly glutamate and GABA (Benes and Berretta, 2001; Tsai and Coyle, 2002), the DAergic hypothesis still affords the best-validated theoretical framework for this disorder. Recent imaging and post-mortem studies have lead to a refinement of this hypothesis, indicating that the dysregulations of DA neurotransmission in cortex and elevations in presynaptic DA content in the striatum may be the main biological signatures of psychotic disorders (Howes and Kapur, 2009; Fusar-Poli et al., 2011; Howes et al., 2011, 2012, 2013; Allen et al., 2012; Egerton et al., 2013; Stokes et al., 2013; Lataster et al., 2014; see Kuepper et al., 2012 and Smieskova et al., 2013 for more thorough reviews on dopaminergic dysfunctions in brain imaging studies in schizophrenia). The increase in presynaptic striatal DA may disrupt informational salience and help contribute to other schizophrenia symptoms (Rosier et al., 2013; Winton-Brown et al., 2014).

The bulk of the evidence suggests that the DAergic deficits in schizophrenia are underpinned by functional, rather than constitutive, abnormalities. Indeed, the majority of studies on post-mortem tissues have failed to identify consistent alterations in the expression of DAergic targets (Harrison, 2000). Accordingly, multiple large-scale genetic analyses have found no robust association for DAergic genes and schizophrenia (Hoogendoorn et al., 2005; Alvarez et al., 2010), and instead point to a predominant involvement of glutamatergic targets (Collier and Li, 2003). In contrast with this evidence, the notion of functional dysregulation of DAergic circuits in schizophrenia is strongly supported by neuroimaging findings, which point to multiple patterns of dysconnectivity between intracortical and subcortical networks (Laruelle, 2003). These dynamic alterations of DAergic neurotransmission are thought to play a key role in the adaptive and neurodevelopmental processes of this system, which are particularly active throughout childhood and adolescence (Teicher et al., 1995; Spear, 2000). These developmental periods may be especially critical for the interactions of DAergic genes with environmental and sex-related vulnerability factors in schizophrenia. In fact, preclinical experiments have shown that sex hormones have a profound influence on the development of the DAergic system throughout early developmental stages (Anderson et al., 2005).

To establish a conceptual framework for the role of DA in schizophrenia, it is necessary to consider that one of the fundamental functions of this system is the extraction of salient information from the environment, through the stimulation of output neurons of cortical and striatal regions integrated within cortico-striato-thalamo-cortical (CSTC) circuits. In particular, the role of mesocorticolimbic DAergic neurons is consistently influenced by the action of glutamatergic and GABAergic cells, which surround and interface with the somata in the ventral tegmental area, as well as the axons and presynaptic boutons in the efferent areas (nucleus accumbens, striatum and PFC). In line with the role of DAergic pathways as neural mediators of informational salience, both the adaptive plasticity and modalities of neurotransmitter release by these neurons are finely regulated by multiple factors; changes in these variables, particularly if occurring during developmental periods, may therefore have long-standing implications on the integrity and coherence of the perceptual process. The modulatory role of DA on processing informational salience is extremely critical during adolescent stages, in which the DAergic system alters cortical innervation and undergoes synaptic maturation and pruning of its glutamatergic and GABAergic connections (Andersen, 2003; O'Donnell, 2010; Burke and Miczek, 2013; Penzes et al., 2013).

The natural corollary of these premises is that the DAergic system may be directly involved in G×S interactions during postnatal development, while prenatal and inborn elements of predisposition may be more directly related to the glutamatergic system (which in turn governs DAergic function through direct and indirect dynamic interactions). This idea is in line with the “multiple hits” hypothesis for the pathogenesis of schizophrenia, which postulates that the disorder may result from the progressive accumulation of deficits from prenatal to juvenile stages, due to different, yet concurring, causes. In the next sections, we will present an overview of the role of sex hormones on schizophrenia, followed by a detailed discussion on the available evidence on the G×S interactions involving DAergic genes.

## The role of sex factors in schizophrenia

### Gender differences in schizophrenia

The existence of gender differences in schizophrenia has been recognized since its first nosographic description by Kraepelin. The best-characterized difference concerns arguably the earlier age of onset in male patients, which typically ranges from 15 to 24 years. In comparison, females exhibit their first overt clinical manifestations between 20 and 29 years, with an average difference of 3–5 years from males (Angermeyer and Kuhn, 1988). It is widely assumed that this divergence in age reflects different developmental trajectories in the DAergic system throughout adolescence across both genders.

A comprehensive and systematic analysis of sex differences in schizophrenia is a complicated undertaking, in view of several methodological issues that may generate spurious results, such as recruitment bias, different metabolic responses to antipsychotic drugs and gender diversity in social adjustment with respect to psychiatric disorders (Markham, 2012). The awareness of these issues, and the numerous discrepancies in the literature have led several authors to cast a skeptical eye on other potential sex differences in schizophrenia, such as prevalence and symptomatic presentation (Häfner, 2002; Jablensky, 2003). Nevertheless, more recent studies, performed with more accurate and tighter controls, have actually found that the gender differences in schizophrenia may encompass several aspects of this disorder, including: (1) a higher risk of schizophrenia in males (~40%) (Markham, 2012); (2) poorer premorbid adjustment in males; (3) a greater severity in clinical course in males, characterized by higher frequency and intensity of negative symptoms, as well as more rapid cognitive deterioration and greater predisposition to relapse (Larsen et al., 1996; Markham, 2012). Current studies investigating the role of sex differences in neuropsychiatric disorders have also highlighted the potential impact of stress and hormonal influences on epigenetic phenomena, which may result in enduring behavioral changes across subsequent generations (see Goel and Bale, 2009; McCarthy et al., 2009; Bale, 2011; McCarthy and Nugent, 2013).

### Role of estrogens in schizophrenia

The prevalent line of interpretation of this sex-related disproportion lies in the neuroprotective role of estrogens in women (Seeman, 1996). Indeed, women display greater severity of their psychotic symptoms in conditions associated with lower concentrations of β-estradiol, the main estrogen hormone, such as fluctuations within the menstrual cycle (Bergemann et al., 2007; Rubin et al., 2010), and menopause (Häfner et al., 1993). Furthermore, plasma β-estradiol levels are reduced in schizophrenia female patients across all phases of the menstrual cycle (Riecher-Rossler et al., 1994), and the age of disease onset in women is inversely related to the age of puberty (Cohen et al., 1999). Accordingly, several clinical trials have shown that additive treatment with estradiol substitutes improves and accelerates the therapeutic response of patients (Kulkarni et al., 1996, 2002; Akhondzadeh et al., 2003; Kulkarni et al., 2008). A number of clinical studies have also shown associations between estrogen receptor polymorphic variants in psychotic-related phenomena (Weickert et al., 2008; Min et al., 2012; Wang et al., 2013). In general, it appears that the sex factors do not induce specific qualitative differences in symptoms, but rather dampen the severity or delay the onset of the same manifestations. The biochemical nature of the neuroprotective effects of estrogens has not been fully qualified yet, but a number of studies point to a direct implication of the DAergic system (Sumner and Fink, 1993; Fink et al., 1998), in addition to glutamate and GABA. In general, the relations between estrogens and DA are supported by a host of clinical and preclinical evidence (for a thorough and detailed presentation of this issue, see Sanchez et al., 2010).

### Role of androgens in schizophrenia

The involvement of sex steroids in schizophrenia is not likely limited to estrogens, but may also include androgen hormones. These steroids appear to exert a multifaceted influence on the neurobiological substrates of schizophrenia; in particular, the complexity of this role stems from the fact that testosterone, the main gonadal androgen, is also converted into β-estradiol via aromatization. Men with schizophrenia tend to exhibit lower levels of testosterone, and testosterone levels are inversely correlated with the severity of negative symptoms (Akhondzadeh et al., 2006; Ko et al., 2007). Furthermore, this hormone has been shown to exert therapeutic properties for negative symptoms in schizophrenia (Ko et al., 2008). In contrast, the role of other androgens in schizophrenia is less clear. For example, schizophrenia patients exhibit high levels of the adrenal androgens dehydroepiandrosterone (DHEA) and androstenedione (Ritsner and Strous, 2010); in addition, DHEA has been found to attenuate the extrapyramidal symptoms induced by antipsychotic drugs (Ritsner et al., 2010).

In general, it is possible that androgenic metabolites of testosterone may facilitate the development of schizophrenia-related symptoms. The conversion of testosterone and androstenedione into their androgenic metabolites dihydrotestosterone (DHT) and androstanedione, respectively, is mediated by 5α-reductase (Paba et al., 2011). Notably, this process competes with the aromatization of the same substrates to β-estradiol and β-estrone. In males, 5α-reductase activity is enhanced during puberty; thus, it is possible that the increased rate of conversion of testosterone and androstenedione into their 5α-reduced androgenic metabolites (instead of estrogens) may contribute to the greater schizophrenia vulnerability and earlier age of onset in males. Our group has tested this intriguing hypothesis in rodent models of schizophrenia; our results indicate that inhibition of 5α-reductase leads to marked anti-DAergic actions on endophenotypes relevant to schizophrenia, such as sensorimotor gating deficits and stereotyped behavior (Bortolato et al., 2008a; Paba et al., 2011; Devoto et al., 2012; Frau et al., 2013). In addition, we recently found that inhibition of another key androgen-synthetic enzyme, CYP17A1, elicits similar, albeit less potent, anti-DAergic effects in the same schizophrenia-related behavioral paradigms (Frau et al., 2014). Collectively, these findings highlight that, in addition to testosterone, other androgens may have a role in the pathogenesis of schizophrenia-related features, through the mediation of DA neurotransmission (for a more detailed description of this issue and its potential therapeutic implications, see Paba et al., 2011).

## The role of DAergic genes in G×S interactions in schizophrenia

### COMT

COMT catalyzes the methylation of the 3O group of catecholamines. The methyl group is donated by S-adenosyl-methionine (SAM), and DA is directly converted by COMT into metanephrine. Other catechol-containing structures are substrates of COMT, including norepinephrine, epinephrine and the DA precursor l-DOPA.

COMT has a soluble form (S-COMT) and a membrane-bound form (MB-COMT), both of which are encoded by the same gene (Lundstrom et al., 1991), located on chromosome 22q11.2. COMT expression is controlled by two promoters in the third exon of the gene (Salminen et al., 1990; Lundstrom et al., 1991). The P1 promoter regulates the expression of a shorter transcript, which can code for S-COMT only (Tenhunen et al., 1993), whereas the more distally located P2 promoter can encode both transcripts. S-COMT is generally dominant in most tissues, with the only exception being in the human brain, where 70% is MB-COMT, and 30% is S-COMT. In the brain, S-COMT is mostly found in the glia and is not likely to serve a primary function in DA metabolism (Rivett et al., 1983; Naudon et al., 1992); conversely, MB-COMT is abundantly localized in postsynaptic terminals of neurons and in perisynaptic locations (Bertocci et al., 1991; Lundstrom et al., 1991; Schott et al., 2010). This form is likely to play a key role in DA degradation, particularly in regions with low DA transporter (DAT) expression, such as the PFC, or, alternatively, in conditions of DAT inhibition (Karoum et al., 1994; Sesack et al., 1998; Huotari et al., 1999; Matsumoto et al., 2003).

**Figure 1:**
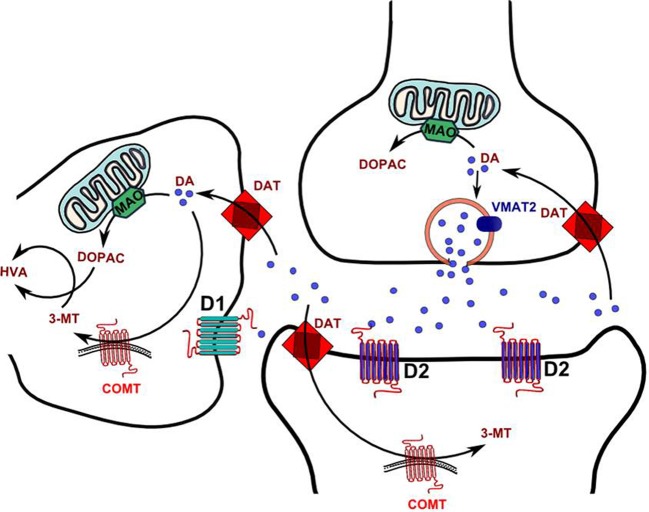
**Schematic diagram of DA synaptic metabolism.** Abbrevations 3-MT, 3-Methoxytyramine; COMT, Catechol-o-methyltransferase; DA, DA; DAT, DA reuptake transporter; DOPAC, 3,4-Dihydroxyphenylacetic acid; HVA, Homovanillic acid; MAO, Monoamine oxidase.

Notably, the effect of COMT on DA metabolism may be particularly dominant in males; indeed, only COMT knockout males exhibit a significant (3-fold) increase in DA levels in the PFC (Gogos et al., 1998). MB-COMT is generally localized intracellularly, but not in the cell membrane (Ulmanen et al., 1997). This distribution implies that its function in DA metabolism is secondary to DA uptake in the postsynaptic terminal (Figure 1), which may be served by either the organic cation transporter 3 (OCT3; SLC22A3) or the plasma membrane monoamine transporter (PMAT; SLC29A4) located in the postsynaptic neuron or glia. PMAT is highly expressed in the forebrain (Engel et al., 2004; Dahlin et al., 2007), including brain regions with sparse DAT expression.

The perisynaptic location of COMT suggests that this enzyme may be important for volume transmission of DA, which plays an important role in the PFC (Paspalas and Goldman-Rakic, 2004). Given the relevance of volume neurotransmission in the PFC for the acquisition of certain informational aspects, such as the perception of salience and the dynamic regulation of signal-to-noise ratio, alterations in COMT activity may result in cognitive changes, particularly with respect to PFC-mediated functions. In addition, COMT may be a crucial element in differentiating the temporal patterns of tonic and phasic DA action (Bilder et al., 2004).

A host of studies have proposed the *COMT* gene as a potential candidate for psychosis and related phenomena (Egan et al., 2001; Williams et al., 2007). In particular, numerous investigations have focused on rs4680, one of the best-characterized single-nucleotide polymorphisms of the *COMT* gene, resulting in the substitution of a valine (*Val*) for a methionine (*Met*) residue at position 108 of S-COMT and 158 of MB-COMT (*Val-Met*) (Lachman et al., 1996; Harris et al., 2005; Wahlstrom et al., 2007). The *Val*-allele confers a higher intrinsic COMT activity than the *Met*-allele (Männistö and Kaakkola, 1999), leading to an overall reduction in DA levels in the PFC. Indeed, COMT serves as the primary enzyme for DA metabolism in this region (Egan et al., 2001; Schott et al., 2006; Tan et al., 2007; Diaz-Asper et al., 2008). Accordingly, individuals harboring the *Val* allele exhibit low DA levels predominantly in the PFC, which may result in a region-specific dysregulation of DA receptors (and particularly D_1_, the most abundant DA receptor in the cortex). Conversely, striatal DA levels and D_2_ receptor availability appear to be unaffected by alterations in COMT activity (Yavich et al., 2007; Hirvonen et al., 2010). Moreover, *Val-*allele carriers have been associated with impaired physiological responses across several functional domains, including cognitive flexibility, working memory, attentional control and emotional resilience (Malhotra et al., 2002; Goldberg et al., 2003; Blasi et al., 2005; Smolka et al., 2005).

The role of COMT in schizophrenia has been extensively studied, yet results have unequivocally shown that neither genetic variants nor the catalytic activity of the enzyme have great intrinsic influence on schizophrenia risk (Chen et al., 1996; Daniels et al., 1996; Riley et al., 1996; Wei et al., 1996; Karayiorgou et al., 1998; Wei and Hemmings, 1999; De Chaldee et al., 2001; Semwal et al., 2001; Strous et al., 2006). Nevertheless, multiple lines of evidence indicate that high-activity *COMT* variants is robustly associated with a greater severity of negative and cognitive symptoms in schizophrenia patients, as well as specific endophenotypic impairments related to functional deficits of the PFC (Egan et al., 2001; Herken and Erdal, 2001; Weinberger et al., 2001; Weinberger, 2002). Specifically, the *Val* allele has been associated with poorer performance in schizophrenia patients across several neuropsychological tests for executive functioning (Goldberg et al., 2003; Nolan et al., 2004; Ohnishi et al., 2006; Diaz-Asper et al., 2008; Opgen-Rhein et al., 2008; Ira et al., 2013), as well as sensorimotor gating deficits in comparison to carriers of the *Met* allele (Quednow et al., 2010). Individuals harboring the *Val* variant also exhibit greater prefrontal noise, corresponding to the electromagnetic activity in the region (Winterer et al., 2006). In contrast, multiple studies have ascertained that the *Met* variant is associated with a slightly lower schizophrenia risk, as well as less severity of attentional, cognitive and information-processing deficits (Egan et al., 2001; Bilder et al., 2002; Bray et al., 2003; Gallinat et al., 2003; Tunbridge et al., 2006; Ehlis et al., 2007; Lu et al., 2007).

Although the aforementioned studies indicate that the *Val* variant confers at best a very modest enhancement of schizophrenia risk, recent investigations suggest that the interaction of this haplotype with other genetic or environmental vulnerability factors may lead to schizophrenia (Schenkel et al., 2005; Stefanis et al., 2007; Collip et al., 2011; Pelayo-Teran et al., 2012). In particular, the interaction of the *Val* variant with cannabis abuse in adolescence has been shown to increase schizophrenia risk (Caspi et al., 2005; Henquet et al., 2006; Estrada et al., 2011), but the neurobiological bases of this interaction remain poorly understood.

While most of the research on *COMT* genotypes and schizophrenia has been focused on the impairments associated with the *Val* variant, emerging lines of evidence have also pointed to the possibility that the *Met*-variant may predispose schizophrenia patients to aggression and violence (Strous et al., 1997; Lachman et al., 1998; Nolan et al., 2000; Liou et al., 2001; Han et al., 2004, 2006; Kim et al., 2008; Tosato et al., 2011; Bhakta et al., 2012; Singh et al., 2012). Interestingly, this predisposition appears to be specific for males, pointing to a potential G×S interaction (Nolan et al., 2000; Soyka, 2011; Singh et al., 2012).

A summary of the main studies that have identified G×S interactions concerning *COMT* polymorphisms is reported in Table 1. Although these data should still be regarded as preliminary, several studies suggest that male patients with high-activity COMT may have greater severity of endophenotypes associated with prefrontal deficits in schizophrenia, such as eye movement disturbances (Rybakowski et al., 2002) prefrontal noise (Winterer et al., 2006) and schizotypal traits (Ma et al., 2007). Similarly, Hoenicka et al. (2010) found that the effects of the *Val*^158^*Met* polymorphism on schizophrenia vulnerability are more directly related to male patients, possibly through an epistatic interaction with D_1_ receptor (Hoenicka et al., 2010) (see below). Other studies indicate that only female carriers of the *Val/Met* alleles exhibit high propensity to engage in risky behaviors (Amstadter et al., 2012) and alterations in emotional processing (Domschke et al., 2012).

**Table 1:** **List of major studies documenting an interaction between COMT polymorphic variants and sex in schizophrenia and related symptoms**.

**Study aim**	**Polymorphisms (SNP if applicable)**	**Total subjects**	**Male: female ratio**	**Finding**	**References**
Clinical response of risperidone in Chinese schizophrenic patients	10 SNPs rs9606186	130	45:85 patients	Increased efficacy of risperidone efficiency in males	Zhao et al., 2012
Gender effects of COMT polymorphisms on cognitive function in children	Val/Val	8707 children	Numbers not indicated	Val/Val genotype scored lower on selective attention and executive functioning than Met/Met in males	Barnett et al., 2007
COMT genetic polymorphisms association with Chinese schizophrenic patients	rs740603 and rs4818	604 (284 patients and 320 controls)	203:81 patients 140:180 controls	Significant association with negative symptoms in females	Li et al., 2012
COMT genotypes in schizophrenia risk	rs165774; rs174675; rs4646316; rs4680; rs6267; rs737866; rs740603	410 (160 patients and 250 controls)	138:22 patients 148:102 controls	Significant genotype assocation with schizophrenia in males	Voisey et al., 2012
COMT polymorphisms association with tardive dyskinesia	rs737865; rs6269; rs 4633; rs4818; rs4680; rs165599	226 (90 positive for Tardive dyskinesia)	140:73 patients	Higher association of antipsychotic-induced tardive dyskinesia occurance in males	Zai et al., 2010
Role of D1 dopamine receptor polymorphisms and its interaction with COMT genotype in schizophrenia	Val/Val	701 (337 patients and 364 controls)	226:111 patients 171:193 controls	D1 dopamine receptor polymorphisms and COMT Val/Val genotype associated with schizophrenia in males	Hoenicka et al., 2010
Impact of COMT genotype on sensorimotor gating in healthy colunteers	Val/Val and Val/Met	107 healthy controls	54:53 controls	Serotonin 2A receptor polymorphisms and males with COMT Val/Val or Val/Met have sensorimotor gating deficits	Quednow et al., 2009
Association between schizotypal traits and COMT in a healthy Chinese population	Val/Met	465 healthy controls	231:234 controls	Met alleles showed increased schizotypal personality questionnaire scores in males	Ma et al., 2007
Association of prefrontal electrophysiologic “noise” and COMT genotype in schizophrenia patients	Val/Val	282 (83 patients; 87 siblings; 112 controls)	65:18 patients 31:56 siblings 66:46 controls	Val/Val-allele males exhibit higher prefrontal “noise”	Winterer et al., 2006
Role of COMT genotype in schizophrenia on performance on wisconsin card sorting test	Val/Val	124 patients	60:64 patients	Male Val/Val alleles displayed best (lowest) scores in Wisconsin card sorting test; Female Val/Val carriers had worst (highest) scores	Rybakowski et al., 2006
COMT genotype on psychosis in Alzheimer's disease	Val/Met	373 patients	130:243 patients	Female Val/Met carriers with Alzheimer's disease had a higher risk of psychosis	Sweet et al., 2005
Role of COMT in schizophrenia vulnerability in Arabic population	Val/Val	332 (255 patients and 77 controls)	161:94 patients 31:53 controls	Female Va/Val carriers display higher risk for schizophrenia, while male Val/Met carriers have higher risk	Kremer et al., 2003
COMT genotype on eye movement disturbances in schizophrenia patients	Met/Met	177 (117 patients and 60 controls)	74:43 patients 29:31 controls	Male schizophrenia patients with Met/Met genotype had lower oculomotor disturbances	Rybakowski et al., 2002
Role of COMT in homicidal behavior in schizophrenia patients	Met/Met	507: 30 violent patients; 62 nonviolent patients; 415 controls	28:2 violent patients 30:32 nonviolent patients 159:256 controls	Higher Met/Met male carriers in violent schizophrenic patients	Kotler et al., 1999
Role of COMT in schizophrenia vulnerability in Jewish population	12 SNPs: Val/Val rs165599 and Val/Val rs165599-rs165688	12906 (2188 patients and 10718 controls)	1383:775 patients 7947:2771 controls	rs165599 Val/Val and rs165599-rs165688 higher in female schizophrenia patients	Shifman et al., 2002
Role of COMT genotype in cognition in children	Haplotype (rs6269; rs4633 and 4s4818) rs2075507 (previously rs2097603); rs6269; rs4818; rs4680; rs165599	8173 children	4211:3962 children	ValB/ValB (lowest COMT activity) haplotype with highest Verbal IQ; Val/Val in rs165599 show lower working memory in males	Barnett et al., 2009

COMT activity has been reported to be higher in males than females (Boudikova et al., 1990). This gender difference may reflect the ability of testosterone and DHT to increase COMT expression (Purves-Tyson et al., 2012). Alternatively, estrogens have been found to reduce the transcription and expression of COMT (Männistö et al., 1992; Xie et al., 1999). An additional mechanism that may predict a lower COMT activity in females may be afforded by the function of catecholestrogens. These 2- and 4-hydroxylated metabolites of β-estradiol (Ball and Knuppen, 1980; Zhu and Conney, 1998) compete with DA for COMT-mediated metabolism, and may act as inhibitors of the enzyme at high concentrations. Accordingly, catecholestrogens have been shown to modulate the turnover of catecholamines (Parvizi and Wuttke, 1983). While the specific role of catecholestrogens on G×S interactions of *COMT* in schizophrenia remains to be investigated, the reduction of COMT activity in females may explain the lower susceptibility of this gender for the phenotypic effects of the *Val* variant on PFC function. At the same time, this mechanism could also account for the higher proclivity of female carriers of the *Met* allele to engage in risky behaviors (Amstadter et al., 2012).

A number of preclinical studies have found sex-specific neurochemical and behavioral differences associated with COMT (Gogos et al., 1998). In particular, heterozygous male COMT-deficient mice exhibit impaired object recognition (Babovic et al., 2008). Conversely, COMT overexpression was found to be associated with blunted stress responsiveness, as well as impairments in working memory and attentional set-shifting (Papaleo et al., 2008). These data are in agreement with evidence showing that COMT alterations may negatively impact prefrontal functions in both humans and rodents (Papaleo et al., 2012). In a recent study, Risbrough and colleagues found that male mice carrying the COMT158*Val*-variant exhibit marked reductions in spatial working memory and disruptions in sensorimotor gating; conversely. female mice carrying the COMT158*Met*-variant displayed alterations in fear-related behavioral responses (Risbrough et al., 2014). Collectively, these preclinical findings further support the role of sex-specific influences of COMT genetic variations on prefrontal DAergic systems.

### MAO A and B

MAO A and B are mitochondrial-bound enzymes (Greenawalt and Schnaitman, 1970) differing by substrate affinity. MAO A has high affinity for serotonin and norepinephrine, while MAO B metabolizes the trace amine phenylethylamine. DA can be degraded by both isoforms; however, the primary enzyme differs across species. In humans and primates, MAO B is the major metabolic enzyme of DA, whereas MAO A serves this role in rodents (Garrick and Murphy, 1980; Cases et al., 1995; Fornai et al., 1999). The metabolism of DA mediated by MAOs occurs, for the most part, in the presynaptic terminal, following reuptake by the DAT. The difference between MAO A and MAO B also concerns their anatomical localization. MAO A is localized in catecholaminergic neurons (and in particular in the locus coeruleus, nucleus accumbens, hypothalamus and mammillary complex), whereas MAO B is found in serotonergic neurons, as well as histaminergic cells and astrocytes (Westlund et al., 1988; Saura et al., 1994; Luque et al., 1995, 1996; Jahng et al., 1997; Bortolato et al., 2008b).

Several studies have documented that *MAOA* gene polymorphisms are associated with different psychiatric disturbances (Ozelius et al., 1988; Black et al., 1991; Hotamisligil and Breakefield, 1991; Hinds et al., 1992; Shih and Thompson, 1999). Most of the genetic studies on MAOA have focused on a variable number tandem repeat (VNTR) polymorphism, which is located 1.2-kilobase upstream of the transcription initiation site and has been associated with changes in gene expression (Sabol et al., 1998). Of the six different allelic variants characterized in humans, the most common display 3 repeats (3R) and 4 repeats (4R) (Sabol et al., 1998; Deckert et al., 1999; Jonsson et al., 2000). The 3R variant has been associated with behavioral features linked to low MAO A activity, such as impulsive aggression and antisocial personality (Oreland et al., 2007; Buckholtz and Meyer-Lindenberg, 2008). In contrast, the 4R variant has been associated with higher *MAOA* gene transcription and enzyme activity (Sabol et al., 1998; Denney et al., 1999). Neuroimaging imaging studies have found a link between the VNTR variants of *MAOA* promoter and structural and functional differences in the PFC (Meyer-Lindenberg et al., 2006).

In general, the majority of genetic studies have failed to find a straightforward association between *MAOA* and schizophrenia (Coron et al., 1996; Sasaki et al., 1998; Syagailo et al., 2001; Norton et al., 2002; Iwata et al., 2003; Li and He, 2008; Wei et al., 2011). Nevertheless, other analyses found preliminary results in support of a sex-specific effect of *MAOA* with respect to schizophrenia diagnosis or select symptomatic aspects of the disorder (Jonsson et al., 2003; Qiu et al., 2009; Camarena et al., 2012; Sun et al., 2012b).

A summary of the main findings on potential G×S interactions involving the *MAOA* gene is reported in Table 2. Although the evidence on sex-dependent effects of MAOA is still preliminary and inconclusive, it was recently reported that male schizophrenia patients exhibit abnormal patterns of methylation of the *MAOA* promoter, pointing to the possibility that the effect of sex may be directly dependent on epigenetic alterations (Chen et al., 2012). In addition to the evidence on schizophrenia, several findings have documented that genetic variations of *MAOA* may play a central role in neuropsychiatric disorders in a sex-dependent fashion. This concept is best highlighted by the elegant study conducted by Caspi and colleagues, showing that males, but not females, harboring low MAO A activity polymorphic variants and subjected to early childhood maltreatment exhibit a significantly higher vulnerability to develop antisocial and aggressive behaviors in adulthood (Caspi et al., 2002; Foley et al., 2004; Kim-Cohen et al., 2006). Indeed, subsequent studies have found that testosterone levels in the cerebral spinal fluid paralleled aggressive responses in carriers of the low MAO A activity polymorphism (Sjoberg et al., 2008). In contrast, DAergic metabolic levels were inversely associated with testosterone in low MAO A activity carriers (Sjoberg et al., 2008). Although both low-activity *MAOA* variants and testosterone have been independently shown to affect aggression, it remains unclear how this genotype may predispose individuals to higher androgen synthesis and how these two properties may interact to influence aggression. It is worth noting that males have a markedly higher frequency of low MAO A activity variants than females (Sjoberg et al., 2008).

**Table 2:** **List of major studies implicating the interaction between MAO polymorphic variants and sex in schizophrenia and related symptoms**.

**Study aim**	**Polymorphisms (SNP if applicable)**	**Total subjects**	**Male: female ratio**	**Finding**	**References**
Role of MAO A and B polymorphisms in negative and positive schizophrenia symptoms	MAOA: 3-repeat and 4-repeat uVNTR MAOB: rs1799863 and rs1137070	468 (344 patients and 124 controls)	209:135 patients 60:64 controls	Higher affective flattening in female schizophrenic patients homozygous for MAOA 4-repeat uVNTR and MAOB/rs1799836 (GG)	Camarena et al., 2012
Role of MAO A gene polymorphisms in paranoid schizophrenia in a Chinese population	MAOA 3-repeat and 4-repeat uVNTR and 41 SNPs	1122 (555 patients and 567 controls)	284:271 patients 308:259 controls	VNTR 3-repeat-rs6323, VNTR 3-repeat-rs1137070 and VNTR 3-repeat-rs6323-rs1137070 haplotypes associated with paranoid schizophrenia in females	Sun et al., 2012a; Sumner and Fink, 1993
Association of MAO A/B genes and schizophrenia in a Chinese population	MAOA: rs6323 MAOB: rs1799836	1073 (537 patients and 536 controls)	294:243 patients 284:252 controls	MAO A rs6323 and MAO B rs1799836 haplotype associated with schizophrenia in females	Wei et al., 2011
Association between antipsychotic-induced restless legs syndrome and MAO polymorphisms in schizophrenia	MAO A: 3-repeat and 4-repeat uVNTR MAO B: A644G SNP	190 patients	106:84 patients	Males patients with MAO A 3-repeat uVNTR and MAO B A644 genotype has higher association with antipsychotic-induced restless leg syndrome	Kang et al., 2010
Association of MAO gene microsatellites with schizophrenia	MAO A: (AC)n repeats MAO B: (TG)n repeats	89 nuclear families with schizophrenic offspring	Not indicated	Families of male schizophrenia patients had higher frequency of transmitted MAO B (TG)_24_ repeats	Wei and Hammings, 1998
Association of MAO A gene variants and schizophrenia in a Chinese population	MAO A uVNTR 3-repeat and 4 repeat and -941G/T and -1460C/T restriction fragment length polymorphisms	355 (234 patients and 121 controls)	156:78 patients 76:45 controls	Haplotype association of schizophrenia with 3-repeat uVNTR and -941T allele in males	Qiu et al., 2009
MAO platelet activity relationship to auditory hallucinations and paranoia in schizophrenics	MAO platelet activity (MAO B)	237 (101 patients and 136 controls)	64:37 patients 65:71 controls	Decreased platelet MAO activity associated with paranoid subtype and presence of auditory hallucinations in male schizophrenia patients	Meltzer and Zureick, 1987

Although the mechanism is unclear, females harboring the high-activity *MAOA* variant display higher baseline cortisol levels than males with the same polymorphism than females carrying low-activity alleles (Jabbi et al., 2007). Furthermore, both females and males harboring low-activity *MAOA* variants exhibited a sexually dimorphic increase in stress response, which was dependent on *COMT* genotype (Bouma et al., 2012). In particular, male carriers of the *Met/Met COMT* allele displayed a significantly higher cortisol response to stress than both females with the same allele and males with other genotypes. Conversely, females carrying the *Val/Val COMT* allele in combination with low-activity *MAOA* variants showed higher stress responses than their male and female counterparts.

Although no common polymorphisms have been reported in the gene's coding region, *MAOB* allelic variants may possess different enzymatic activities (Balciuniene et al., 2002; Costa-Mallen et al., 2005). Indeed, several groups have reported the association of polymorphic variants of *MAOB* gene with several neuropsychiatric disorders characterized by DAergic dysfunction. In particular, *MAOB* allelic variations have been associated with bipolar disorder (Lin et al., 2000) and higher schizophrenia susceptibility (Hovatta et al., 1999; Gasso et al., 2008; Carrera et al., 2009; Piton et al., 2011); these results, however, have been not been consistently replicated (Coron et al., 1996; Sobell et al., 1997; Matsumoto et al., 2004; Bergen et al., 2009).

A direct implication of *MAOB* in schizophrenia is supported by several studies (Coron et al., 1996; Bergen et al., 2009; Carrera et al., 2009; Piton et al., 2011; Wei et al., 2011; Sun et al., 2012a) and may be reflective of the greater contribution of this enzyme to the metabolism of DA in humans. In particular (see Table 2), numerous articles have recently reported that different *MAOB* variants may predispose to schizophrenia in women (Gasso et al., 2008; Wei et al., 2011) or in men (Wei and Hemmings, 1999). In addition, other studies highlighted that *MAOB* variants may moderate several symptomatic aspects of schizophrenia, including flat affect (Camarena et al., 2012) or paranoid manifestations (Sun et al., 2012a). Although little is currently known on the potential interaction of sex hormones with MAO B, females have been reported to display significantly higher MAO B activity in platelets in comparison with males (Snell et al., 2002).

A plethora of studies has shown that sex hormones differentially affect MAO activity and expression in specific brain regions. Androgens increase MAO transcription in the substantia nigra (Ou et al., 2006; Purves-Tyson et al., 2012) and in the striatum (Thiblin et al., 1999). Chronic administration of anabolic androgenic steroids, however, reduces MAO activity in the caudate and amygdala, as well as DA metabolites in the nucleus accumbens shell (Birgner et al., 2008). Similarly, gonadectomy also increases MAO A activity in the PFC (Meyers et al., 2010), suggesting that acute treatment with androgens may enhance MAO activity, while chronic treatment exerts the opposite effect. In contrast to androgens, estrogen administration to neonatal, but not adult males elicits an increase in hypothalamic MAO activities (Vaccari et al., 1981). In females, estradiol reduces MAO A activity in the hypothalamus and amygdala (Luine et al., 1975; Ma et al., 1993; Gundlah et al., 2002).

### Other DAergic targets

The current evidence on the implication of the other DAergic targets in G×S interactions is scant and mostly limited to DAergic receptors. Interestingly, several studies have shown that polymorphic variants of the genes encoding D_1,_ D_2_, and D_4_ receptors are linked to different responses to antipsychotic medications in a gender-sensitive fashion. For example, variants of the *DRD2* gene, which codes for the D_2_ receptor, may predispose females to a greater prolactin increase in response to antipsychotics (Mihara et al., 2000, 2001); however, this difference may not be dependent on an actual G×S interaction, but rather on the higher baseline levels of prolactin in females (Yasui-Furukori et al., 2008). Variants of the *DRD1* and *DRD4* genes (coding for D_1_ and D_4_ receptors) may also predispose to different responses to antipsychotic treatment (including side effects) (Hwu et al., 1998; Potkin et al., 2003; Hwang et al., 2007; Popp et al., 2009). Only few studies have pointed to a direct role of these genes in specific symptomatic aspects. Different variants of the *DRD2* gene may be associated with higher perseverative responses in female schizophrenia patients (Rybakowski et al., 2005), while VNTR variants of the *DRD4* gene may predict for differences in age of onset in female patients (Goncalves et al., 2012). Notably, the *DRD1* gene has been recently found to establish an epistatic interaction with the *COMT* gene, which predicts schizophrenia risk in males, presumably due to the functional association of D_1_ receptors and COMT in the PFC (Hoenicka et al., 2010).

Independent investigations have reported that different variants of the *DRD3* gene may be associated with schizophrenia predisposition in males (Asherson et al., 1996; Griffon et al., 1996) and females (Aksenova et al., 2004; Godlewska et al., 2010). Interestingly, preliminary results in our animal models suggest that the behavioral responses elicited by agonists of D_3_, but not D_2_ receptors, may be under control of neurosteroids with respect to the regulation of sensorimotor gating (Frau et al., submitted). Future work is warranted to establish the nature of this intriguing neurobiological finding.

## Concluding remarks

As mentioned above, the attempts to identify a genetic basis of schizophrenia have revealed a picture of extreme complexity and high heterogeneity of heritable bases. This view has gradually replaced our “genome-centric” perspective with a broader framework, in which genetic vulnerability is a piece of a much greater mosaic, consisting of complex interactions with environmental factors. In this perspective, sex hormones may also play a significant role in shaping the course of schizophrenia and modifying the developmental trajectory of the neurobiological alterations of DA and other neurotransmitter systems underpinning this disorder.

The findings summarized in this review indicate that, although the role of G×S interactions in schizophrenia is still inconclusive, sex hormones might affect brain substrates through a multilayered set of mechanisms, which appear to have a particular impact on the catabolic apparatus of DA.

The diagnostic definition of schizophrenia (as based on the DSM-IV and DSM-5) is only related to symptomatic descriptors, rather than biomarkers and quantitative endophenotypes. This scenario raises the possibility that this disorder may actually correspond to an array of diverse clinical conditions which share a common “final pathway” accounting for the pathognomonic manifestations of schizophrenia. Accordingly, a greater understanding of the role of DA neurotransmission in schizophrenia may have important repercussions also with respect to a better nosographic classification of this disorder.

The integration of preclinical research with neuroimaging and genetic studies will play a critical role in enabling us to identify central neurobiological networks that underpin gender-specific neurobehavioral endophenotypes of schizophrenia. Additionally, the contribution of these studies and a greater understanding of sex-dependent epigenetic mechanisms of transcriptional regulation will be fundamental to qualify premorbid signs and symptoms, and chart the developmental trajectory of psychosis in males and females.

### Conflict of interest statement

The authors declare that the research was conducted in the absence of any commercial or financial relationships that could be construed as a potential conflict of interest.
